# The interpretation of COVID-19 in cause-of-death statistics: a matter of causality

**DOI:** 10.3205/id000087

**Published:** 2024-09-11

**Authors:** Peter P. M. Harteloh

**Affiliations:** 1 The Hague, The Netherlands

**Keywords:** COVID-19, underlying cause of death, death certificate, cause-of-death statistics, health policy

## Abstract

**Background:**

Mortality is an important indicator for estimating the impact of the COVID-19 pandemic. However, different registrations provide different figures and the question is how to interpret the number of COVID-19 deaths reported.

**Objective:**

To study the role of COVID-19 in dying in order to explain the representation of COVID-19 in cause-of-death statistics.

**Methods:**

Analysis of all death certificates mentioning COVID-19 in the Dutch cause-of-death registry during the pandemic (n=51,181). The role of COVID-19 as cause of death was studied by the way it was reported on death certificates. A calculation of odds ratios was performed for studying associations between COVID-19 and other reported causes of death.

**Results:**

In 24% of the cases COVID-19 was the only cause of death mentioned on a death certificate. In 76% of the cases, one or more other diseases played a role in dying. Three patterns emerged: COVID-19 associated with 1. neurodegenerative disorders, 2. chronic respiratory disorders, and 3. metabolic disorders. Of all death certificates mentioning the diseases, COVID-19 was the start of the causal chain leading to death in 45.2% of the cases, while COVID-19 was selected for cause-of-death statistics by special World Health Organization WHO instructions in 93.9% of the cases.

**Conclusions:**

Cause-of-death statistics overestimate the role of COVID-19 as underlying cause of death. In a majority of the deceased cases, there is an association of COVID-19 with other diseases not captured by cause-of-death statistics reporting (only) one cause of death per deceased. A multi-causal approach is needed to evaluate the pandemic and inform health policy.

## Introduction

On 27^th^ February 2020, the first patient with the “Corona Virus Disease 2019” (COVID-19) was reported in the Netherlands [1]. It was the beginning of an epidemic that would rage to November 2022 when the National Institute of Public Health (RIVM) – holding the obligatory registration of infectious diseases – declared the end of the epidemic in The Netherlands [2]. Although the virus still circulated in the Dutch population, its virulence as indicated by symptoms, hospital admissions, intensive care occupancy and mortality had decreased so much that anti-COVID-19 policy measures could be abolished. The RIVM registration of infectious diseases reported almost 23,000 COVID-19 deaths over the period of 2020–2022, while according to the cause-of-death registration of Statistics Netherlands (CBS) about 48,000 deaths were attributed to COVID-19 [3], [4]. There is no easy explanation for this (large) difference. The RIVM registration is obligatory by law and the CBS registration covering all deceased residents of the Netherlands follows international prescribed rules for classifying deaths by the ICD-10 [5], [6]. Confusion is increased by the World Health Organization (WHO) ranking the Netherlands in their country report on COVID-19 deaths by the RIVM figures, not by the CBS figures [7]. This raises the question how to interpret the number of COVID-19 deaths in cause-of-death statistics. An understanding of the death toll is important for estimating the impact of the pandemic or evaluating health policy measures. Therefore, this study investigates the role of COVID-19 as cause of death and its statistical representation.

## Material and method

**Study material:** The role of COVID-19 as cause of death was studied by death certificates. A death certificate enables a physician to describe a causal sequence of events leading to death and express the role of a disease as cause of death – direct, intermediate, underlying or contributory – from a clinical point of view [8]. In the Netherlands, a death certificate is issued by an attending physician and processed by Statistics Netherlands for the production of cause-of-death statistics. At Statistics Netherlands, all causes of death mentioned on a death certificate are coded and the underlying cause of death – the starting point of the causal sequence of events that led to death – is selected for statistical purpose by international (WHO) prescribed rules [5]. During the years 2020–2022, death certificates were processed automatically by Iris – a software incorporating the international rules – version 5.6 [9], [10]. All death certificates mentioning COVID-19 were reviewed manually by medical coders to check the application of the rules and interpret the notation of COVID-19 on a death certificate. COVID-19 was coded according to the WHO special instruction issued in April 2020 [6]. Analysis included all death certificates with a natural cause of death (n=466,538) in 2020–2022, so that death certificates mentioning COVID-19 (n=51,181), regardless whether the disease was selected as underlying cause of death or not, could be compared with death certificates not mentioning COVID-19 (n=415,357). 

**Method:** The study included all causes mentioned on death certificates. Reported external signs of death (respiratory or cardiac arrest, shock) and ill-defined causes of death as listed by WHO were removed [5], [11]. Then the number of (meaningful) causes per deceased was studied. A death certificate mentioning only one disease (with or without complications) should – if completed correctly – indicate a necessary and sufficient condition for death, for instance: a severe infectious disease without therapeutic options. A death certificate mentioning more than one disease is reporting a cluster of diseases leading to death, for instance: an infectious disease in combination with a compromised immunological resistance by a cancer (treatment). Death certificates reporting recognized manifestations of COVID-19 (listed in footnote of Table 1 (Tab. 1)) in addition to the diagnosis “COVID-19” were considered to report one cause of death. The WHO coding instructions enabled to distinguish a clinical or laboratory confirmed COVID-19 (ICD-10 code: U07.1) from a suspected COVID-19 (ICD-10: code U07.2) [6]. 

Death certificates with COVID-19 on the lowest used line were identified in order to compare the regular ICD-10 principle of selecting one underlying cause of death per deceased for statistics with the application of the WHO special instruction for selecting COVID-19 as underlying cause of death [5], [6]. In accordance with the ICD-10 manual, the lowest used line was defined by the actual position of COVID-19 on part 1 of the death certificate while there were no obvious causes on part 2 of the death certificate (direct sequela) overruling its role as underlying cause of death [5]. For applying this ICD-10 principle to COVID-19, the list of obvious causes for a pneumonia has been used as it is the major manifestation of COVID-19 [5].

In order to study the relationship of COVID-19 with other causes of death, odds ratios (OR) were calculated representing the chance of a particular cause of death occurring on a death certificate mentioning COVID-19 versus the chance of that particular cause of death occurring on a death certificate not mentioning COVID-19 [12]. The OR was standardized by the age distribution of the deceased not mentioning COVID-19 as cause of death, so that a difference in observed OR cannot be biased by a difference in population structure. A 95%-confidence interval was calculated to test the statistical significance of an observed association [12]. 

**Ethics:** According to Dutch Civil Law (Article 7: 458) no ethical approval is required for a secondary analysis on non-identifiable data of deceased persons. 

## Results

**Figure 1:**
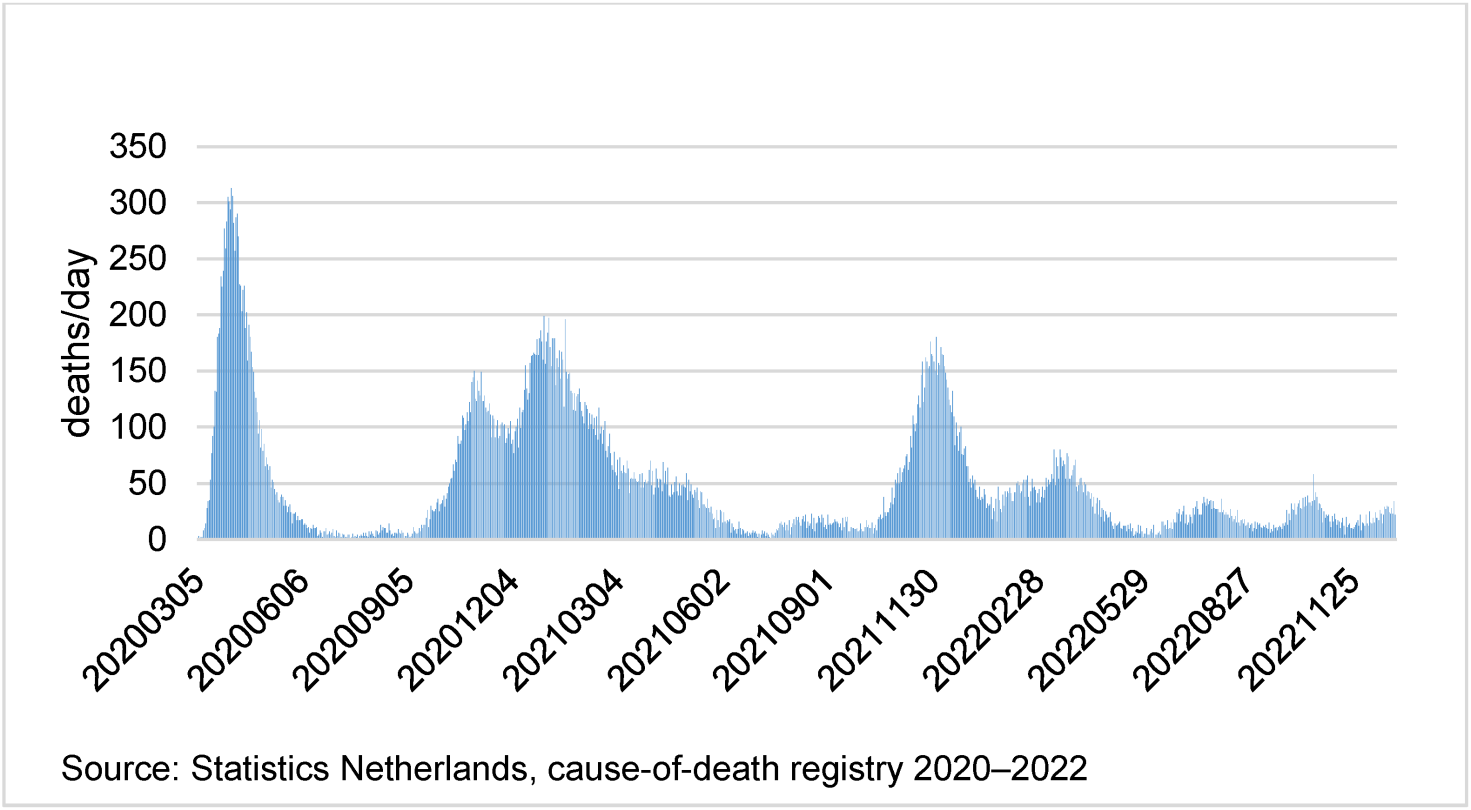
Mortality of COVID-19 in the Netherlands (2020–2022)

The COVID-19 pandemic hit the Netherlands in three mortality waves (Figure 1 (Fig. 1)). The first mortality wave lasted from March to June 2020, the second from September 2020 to June 2021; the third mortality wave started in November 2021 and showed various gradually diminishing outbreaks during 2022. The first wave was caused by the original Wuhan variant of SARS-CoV-2. The second wave was two-, maybe even three-headed, indicating different variants of the virus (Beta and Delta). The third wave was caused by different variants of the Omicron variant of SARS-CoV-2. 

According to the CBS cause-of-death registration, the first wave counted 10,460 deaths mentioning COVID-19 (15.7% of all deceased during the first wave), the second wave 22,117 (16.9% of all deceased during the second wave), and the third wave 17,554 (8.4% of all deceased during the third wave). The remaining deaths occurred in between these waves amounting up to a total of 51,181 COVID-19 deaths in 2020–2022 (11% of all deceased during 2020–2022). According to the WHO special instructions for coding COVID-19 issued April 2020, 48,042 (93.9%) of these cases were attributed to COVID-19 as underlying cause of death and reported in cause-of-death statistics.

Table 1 (Tab. 1) shows the number of causes reported on death certificates. When COVID-19 was mentioned as cause of death, 24.4% of the deaths were attributed to COVID-19 alone. The average number of causes reported was 2.6 and 75.6% of these deaths were caused by one or more other diseases in addition to COVID-19. For deaths without mention of COVID-19, the mean number of causes was 2.7. Most of these deceased (75%) died with 1–3 causes of death. The number of causes did not differ significantly for death certificates with or without mention of COVID-19. 

Table 2 (Tab. 2) shows the role of COVID-19 as cause of death in the way it was reported on death certificates by attending physicians. The first three columns show the WHO prescribed format of a death certificate used in countries all over the world [5]. There is a part 1 for reporting the causal sequence leading to death and a part 2 for diseases without which the patient would not have died, but that were not part of a causal sequence (contributory causes of death). A direct cause of death (if reported) should be noted on part 1a, an intermediate cause of death (if any) on part 1b, and the underlying cause of death is to be found on the lowest used line in part 1, i.e. part 1c in case of a fully completed death certificate or part 1a or 1b in case of no direct or intermediate cause (reported) [8]. The connection between diseases on different lines of part 1 should be causal (due to) as the second column of the death certificate indicates. The columns 4–7 of Table 2 (Tab. 2) inform about the position of COVID-19 observed on death certificates in the Netherlands during the pandemic: as underlying cause of death on the lowest used line, mentioned with other causes of death, and being clinical/laboratory confirmed or suspected. 

In 45.2% of the cases, COVID-19 was reported on the lowest used line of the death certificate (records with just one cause/only COVID-19 included): 25.2% on part 1a, 14.6% on part 1b and 3.6% of the cases on part 1c. In 1.8% of the cases (even) part 2 – the position for a contributory cause of death – was the lowest used line of the death certificate, implying part 1 did not contain a well-defined cause of death or only symptoms. 

When COVID-19 was mentioned on a death certificate with other causes of death (75.6% of all mentioned), it was noted on part 1a in 38.1% of the cases – implying another disease as underlying cause of death – and on part 1b in 24.0% or part 1c in 6.1% of the cases – implying complications or other conditions (e.g. bacterial pneumonia) due to COVID-19 leading to death. In 7.4% of the cases, COVID-19 was reported as contributory cause of death. 

Of all death certificates mentioning COVID-19 in 2020–2022, 93.4% reported a clinical or laboratory confirmed COVID-19 (ICD-10 code: U07.1) and 6.6% a suspected COVID-19 (ICD-10 code: U07.2). The percentage of suspected COVID-19 cases decreased sharply with the increase of test facilities from 12–30% during the first mortality wave to less than 1% after June 2020. 

Table 3 (Tab. 3) shows the combinations of causes reported on death certificates mentioning COVID-19 by the number of causes reported. COVID-19 was mainly associated with different clusters of dementia (dementia plus cachexia and/or dehydration) and with chronic obstructive pulmonary disease (COPD), heart failure or diabetes mellitus whether or not in combination with dementia. 

Table 4 (Tab. 4) shows the frequency of occurrence of (other) causes on death certificates with and without a mention of COVID-19 (regardless whether the disease was underlying cause of death) and the OR of COVID-19 coinciding with another cause of death. The co-occurring causes are listed in order of decreasing frequency by actual and standardized (in between brackets) numbers for specific and two major (ICD-10) group of causes. 

There was a statistically significant association (lower border 95%-confidence interval >1.0) between COVID-19 and dementia, diabetes mellitus, COPD, asthma, Parkinson’s disease and obesity. Heart failure, stroke, hypertension, chronic ischemic heart disease, and the groups of cardiovascular diseases or cancers occurred statistically less significant in deceased with COVID-19 than in deceased without COVID-19.

## Discussion

Cause-of-death statistics are based on death certificates providing information on the role of COVID-19 as cause of death from a clinical point of view. In 24% of the cases, death certificates in the Netherlands show COVID-19 as the only cause of death, indicating a relatively short, fulminant course of the disease leading to serious complications and death. For a relatively new disease such as COVID-19, there may be an underreporting of death related co-morbidity as pathophysiological connections are not (yet) recognized or death certificates are not filled in completely, because of the high work pressure in hospitals and nursing homes during the pandemic. However, other studies also found 25–28% of the death certificates mentioning only COVID-19 as cause of death [13], [14]. In addition, a meta-analysis showed that close to one-quarter of the individuals with COVID-19 developed a severe disease course [15]. These findings are in line with the results of this study and confirm the idea that COVID-19 is a necessary and sufficient cause of death in at least one quarter of the deceased cases.

In 76% of the cases, COVID-19 was reported as part of clusters causing death. According to contemporary causal theory, COVID-19 is a “sufficient, but not necessary” cause of death in these cases [16]. Three patterns emerged. First, there is an association between COVID-19 and neurodegenerative disorders. This association is confirmed by other studies [13]. However, it is not established (yet) if the explanation is to be found in the living situation of elderly persons or in pathophysiological mechanisms. Neurodegenerative disorders like dementia or Parkinson’s disease increase the risk of contracting a SARS-CoV-2 infection due to the behavioral patterns of patients and their living situations (nursing homes). In addition, aging involves subclinical chronic inflammation (“inflame-aging”, IL-6 elevation) and the gradual development of an acquired immune-senescence, regardless of the presence of co-morbidity [17], [18]. As a result, there could be an increased risk of a serious, i.e. a lethal course of COVID-19 in patients with neurodegenerative disorders. 

Second, there is an association between COVID-19 and the metabolic syndrome, represented by diabetes mellitus and obesity in this study. In the metabolic syndrome there is a reduced function of ACE2 receptors, i.e. the receptor that SARS-CoV-2 uses to enter the cell. This is thought to be the reason why the SARS-CoV-2 infection is more likely to be fatal in this condition than in people without the metabolic syndrome. In addition, a hyperglycemia accompanying (a poorly controlled) diabetes could promote virus replication [19]. In this study, obesity was strongly associated with diabetes showing it as part of the metabolic syndrome. However, as an independent risk factor obesity may also play a causal role in death due to the decreased lung function (i.e. capacity) and the chronic subclinical inflammation indicated by elevated cytokine concentrations in blood and tissues [20].

Third, there is an association between COVID-19 and chronic respiratory diseases, such as COPD and asthma. The ACE2 receptor is present in the lungs, tongue, the tissue that lines the intestines and on endothelial cells including blood vessels. Due to the chronic inflammation of the lung in COPD or asthma, the number of ACE2 receptors in the lung increases. This makes the lung tissue more vulnerable to a SARS-CoV-2 infection. The SARS-CoV-2 also produces cytokines that reduce ACE2 receptor function and promote inflammation and vascular permeability in the lung [21], [22].

These pathophysiological mechanisms may explain the clusters of causes reported on death certificates. Death certificates show the clinical reasoning of physicians observing the death of a patient with COVID-19, a reasoning in line with the general idea of an infectious disease as an interplay between a micro-organism with its virulence, the host with immunological defense mechanisms depending on age and co-morbidity, and the environment with its therapeutic or preventive measures. 

In case of COVID-19 deaths, other studies reported co-morbidity too [14], [23]. However, they did not compare death certificates in a standardized way which is probably the reason why this study could not confirm the association reported by other studies between cardiovascular diseases or cancers and COVID-19 as cause of death. Cardiovascular diseases, cancer or dementia may be present at the end of life, but they do not have to play a role as cause of death, i.e. from a clinical point of view the patient would not have died without the disease being present [8]. A *co-occurrence*, i.e. diseases being present at the end of life while not being a cause of death, and what could be called *co-mortality*, i.e. clusters of diseases causing death, should be distinguished. 

**Statistical representations:** The clustering of COVID-19 with other causes of death poses a problem for cause-of-death statistics. On average death certificates with COVID-19 contain 2.6 causes of death as shown in this and other studies [14], [24], [25]. This implies selection for representation of a case in a cause-of-death statistics based on (just) one cause per deceased [24]. In principle, the disease which is the start of the causal sequence of pathophysiological events leading to death is selected [5]. This starting point is considered object of interventions (prevention or therapy) inhibiting death. This study shows COVID-19 as start of the causal chain in 45.2% (approximately 23,000) of the death certificates mentioning the disease. However, the special WHO instruction prescribes to select the disease for cause-of-death statistics in every deceased mentioning COVID-19 in part 1 of the death certificate, regardless whether it was the start of the causal chain or not [6]. Due to this instruction, COVID-19 was selected for cause-of-death statistics in 94% (48,042) of the death certificates mentioning the disease, an estimate about twice as large as an interpretation of the death certificate incorporating the certifier’s clinical point of view would yield. 

The special WHO instruction for reporting COVID-19 in cause-of-death statistics was motivated by surveillance [6]. Clinical causal considerations are overruled by a health policy perspective. However, for surveillance other sources like the obligatory registration of infectious disease can also be used. They show the same pattern of COVID-19 outbreaks as cause-of-death statistics and report a number of COVID-19 deaths (23,000) that comes close to the clinical estimate of COVID-19 as underlying cause of death [2], [3]. Also, the surveillance should include co-mortality, as in the majority of deceased cases COVID-19 is associated with another cause of death. Current cause-of-death statistics based on one death per deceased fail short in doing this. The observed clusters of co-mortality call for interventions not only being aimed at COVID-19 as such, but at the care for dementia, the treatment of COPD and the prevention of the metabolic syndrome as well in order to prevent death in case of COVID-19. 

**Strengths and limitations:** The strength of this study lies in the material studied: an investigation of the role of COVID-19 as cause of death by death certificates incorporating the clinical judgment of physicians with regard to the death of their patient. Such a judgement might differ from the findings at autopsy, but it stands out as a source of information on its own with a particular view on causality, namely in case of an infectious disease like COVID-19 multi-causality instead of mono-causality, as “the” cause of an infectious disease (virus, host factors, environment) requires [25], [26].

Also, in their WHO prescribed format, the number of causes reported, and the age or sex structure of the deceased, death certificates in the Netherlands do not deviate from those of other high-income countries, so that our sample and findings can be considered representative of COVID-19 deaths in comparable countries. Currently, there are no other bridge coding studies available comparing the usual ICD-10 principles with the special WHO instruction for selection of COVID-19 as underlying cause of death for statistics on the same data set. Therefore, the findings of this study require replication by others in order to gain validity. 

A limitation of this study is the proper completion of a death certificate. The certifier will have to determine for each disease or condition present at the end of life or in the patient’s history whether the patient *would* have died if (s)he would *not* have had the disease or condition (counterfactual) [8], [27]. However, applying the counterfactual is more difficult for a new disease like COVID-19 than for diseases with a well-known pathophysiological mechanism. Underreporting of co-mortality has to be considered despite the corresponding findings of different studies on death certificates. Therefore, linking the cause-of-death registry to other registries is recommended. By linking the cause-of-death registry with clinical registrations, insight can be obtained in co-mortality underlying death and the role of a disease as cause of death can be further investigated [28].

## Conclusions

For the interpretation of COVID-19 in cause-of-death statistics a necessary and sufficient cause of death should be distinguished from a cluster of diseases causing death. In a majority of deceased cases (76%) there appeared to be a statistically significant association between COVID-19 and dementia, Parkinson’s disease, COPD, asthma, diabetes mellitus and obesity. These diseases are associated with a reduced resistance/immunological defense, or promote infection by a cytokine storm or increased number of ACE2 receptors. Cause-of-death statistics – based on one cause per deceased – are not able to capture these dynamics. They serve surveillance from an epidemiological point of view, but overestimate the number of COVID-19 deaths from a clinical (causal) point of view. 

## Notes

### Short author biography

Peter Harteloh worked as medical epidemiologist on cause of death statistics for Statistics Netherlands for 17 years. He is retired now.

### Author’s ORCID

Peter Harteloh: 0000-0001-6271-3813

### Disclaimer

The opinions expressed in this article are of the author and do not necessarily represent the views of Statistics Netherlands.

### Ethics

There were no living persons involved in this study. The analysis is based on data not reducible to individual persons.

### Competing interests

The author declares that he has no competing interests.

## Figures and Tables

**Table 1:**
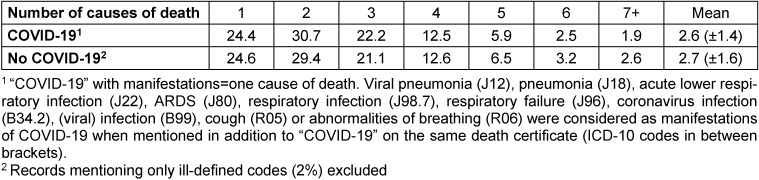
Number of causes on death certificates with (n=51,181) and without (n=407,358) mention of COVID-19 (percentages, mean and standard deviation)

**Table 2:**
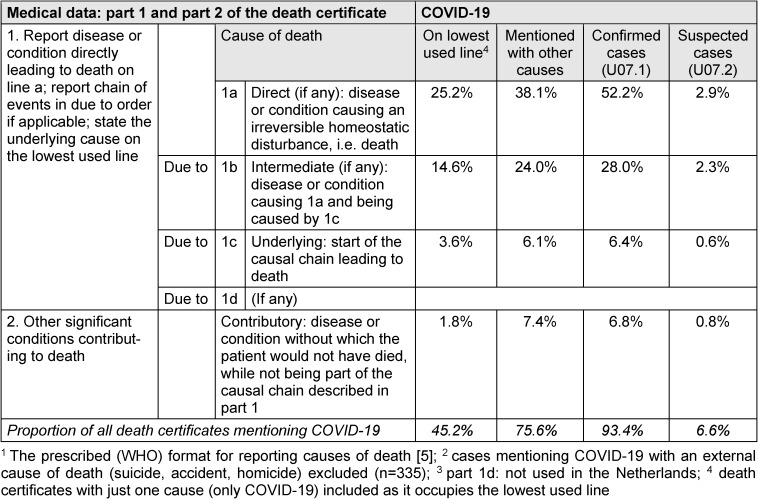
The role of COVID-19 as cause of death when mentioned on a death certificate (n=51,181)^1, 2, 3^

**Table 3:**
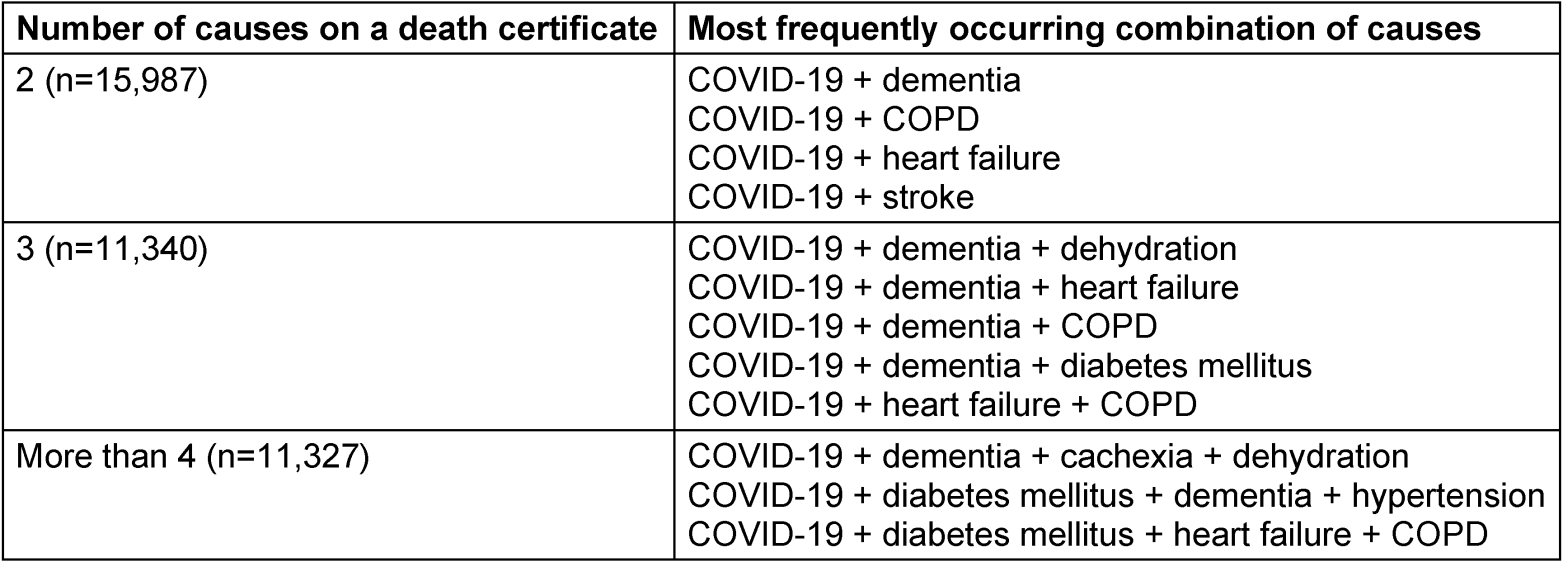
Patterns of co-mortality on death certificates mentioning COVID-19

**Table 4:**
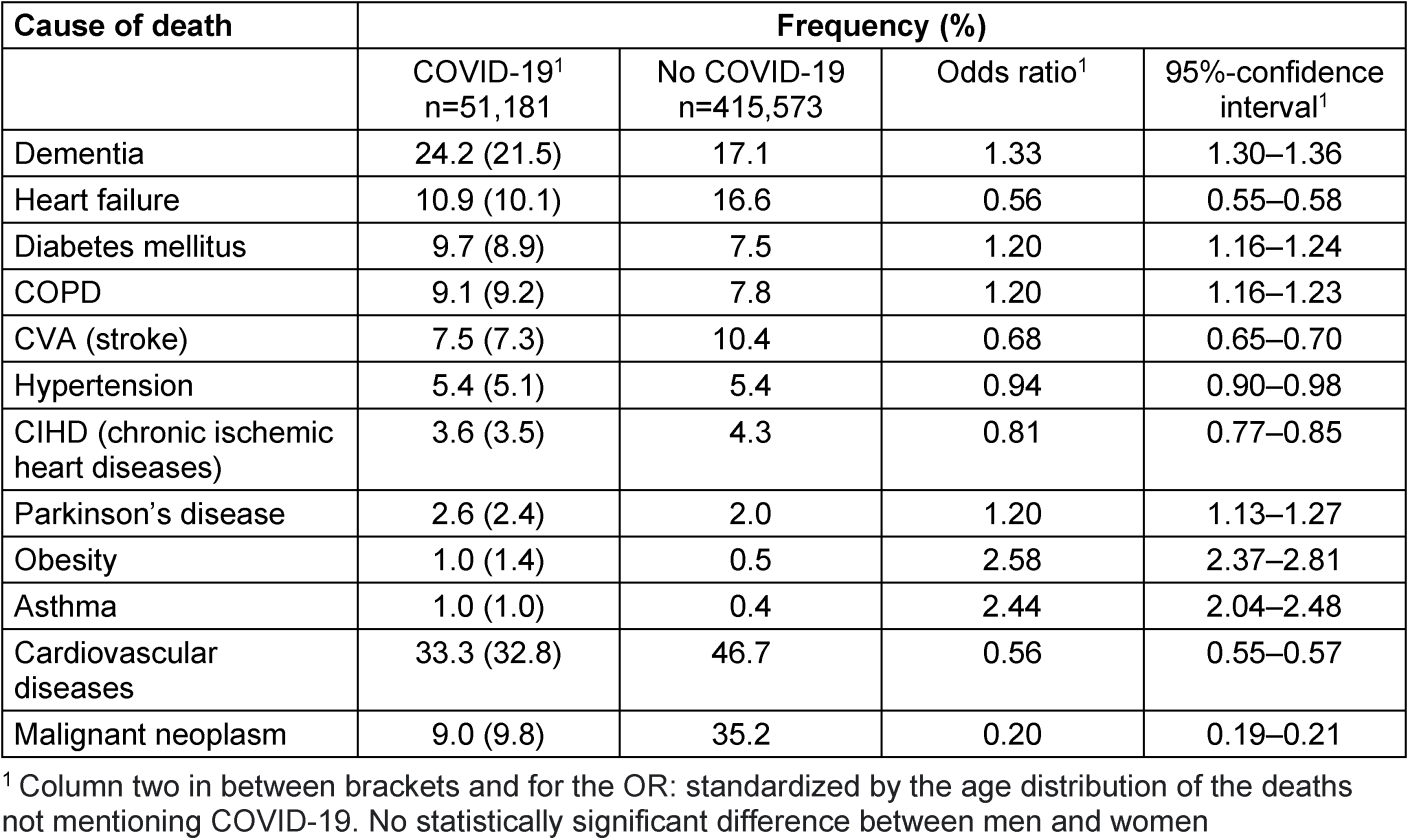
Co-mortality of deceased with or without COVID-19 in the Netherlands
